# Molecular Epidemiological Investigation of Salmonid Alphavirus Genotype 2 (SAV2) Emergence on the Helgeland Coast of Norway in 2023

**DOI:** 10.3390/v18101089

**Published:** 2026-10-02

**Authors:** Jan Inge Øvrebø, Hilde Sindre, Hege Løkslett, Torfinn Moldal, Monika J. Hjortaas

**Affiliations:** Norwegian Veterinary Institute, 1433 Ås, Norway; hilde.sindre@vetinst.no (H.S.); hege.lokslett@vetinst.no (H.L.); torfinn.moldal@vetinst.no (T.M.); monika.hjortaas@vetinst.no (M.J.H.)

**Keywords:** salmonid alphavirus, *Alphavirus salmon*, SAV2, panchreas disease, genomic epidemiology, aquaculture

## Abstract

Salmonid alphavirus (SAV) causes pancreas disease (PD) in Atlantic salmon and is a major viral pathogen affecting salmon aquaculture in Northern Europe. In Norway, PD is associated with genotypes SAV3 and marine SAV2, which have distinct geographical distributions. SAV3 predominates along the southwestern coast, whereas SAV2 has historically been restricted to Mid-Norway. In 2023, SAV2 was detected at four aquaculture sites along the Helgeland coast, north of the established PD zone, representing substantial northward expansion of the virus’ distribution. Whole-genome sequencing (WGS) provides high-resolution data for molecular epidemiological investigations and can distinguish between local viral persistence and recent introductions. In this study, nanopore-based WGS was applied to SAV2-positive samples collected from Norwegian aquaculture sites between 2017 and 2025. Genome sequences were analyzed with previously published Norwegian SAV2 sequences using Bayesian phylogenetic methods. Phylogenetic analyses estimated the most recent common ancestor of the Norwegian SAV2 lineage around 2006, indicating that the virus likely circulated in Norway for several years before its initial detection. SAV2 genomes from Helgeland showed high genetic similarity to genomes from sites within the PD zone, providing strong evidence of aquaculture-related spread. These findings demonstrate the value of WGS for tracing transmission pathways and supporting PD outbreak investigations.

## 1. Introduction

*Alphavirus salmon*, commonly referred to as Salmonid alphavirus (SAV), is the etiological agent of pancreas disease (PD) in Atlantic salmon (*Salmo salar*) and sleeping disease (SD) in rainbow trout (*Oncorhynchus mykiss*) [1,2,3]. SAV is an enveloped, positive-sense single-stranded RNA virus belonging to the family *Togaviridae* and was the first alphavirus described in fish [3,4]. SAV possesses a ~12 kb genome comprising two open reading frames (ORFs) that encode a non-structural polyprotein (8 kb) and a structural polyprotein (4 kb) [3,4,5]. Translation of the non-structural proteins occurs from the genomic RNA, whereas the structural proteins are translated from a polyadenylated 4 kb subgenomic RNA. Salmonid alphavirus genotype 1 (SAV1), initially named salmon pancreas disease virus (SPDV), was the first genotype to be identified [2], followed by SAV2, initially named sleeping disease virus (SDV) [6]. The subsequent identification of a Norwegian SAV strain led to the designation of SAV3. Thus, the first three recognized SAV genotypes were SAV1, SAV2, and SAV3 [7]. Subsequently, four additional genotypes, SAV4, SAV5, SAV6 and SAV7, were described [8,9]. The marine SAV genotypes, afflicting Atlantic salmon, exhibit a degree of geographical separation. SAV1, SAV4 and SAV6 have been reported in Ireland, whereas SAV1, SAV2, SAV4 and SAV5 have been reported in Scotland [10]. In Norway, disease outbreaks are exclusively associated with SAV2 and SAV3, which display distinct geographical distributions [11].

Pancreas disease in Atlantic salmon is characterized by degeneration and loss of pancreatic tissue together with lesions in the heart and skeletal muscle [1]. Affected fish typically exhibit reduced feed intake, lethargy, impaired growth and variable mortality, often followed by prolonged recovery periods in surviving stocks [12,13]. PD was first recorded in farmed Atlantic salmon in Scotland in 1976 [14]. The disease was subsequently reported in the United States [15], Norway [16] and Ireland [17], before the causative virus was first characterized in 1995 [2]. The disease has since become one of the most significant viral challenges in European salmon aquaculture and is associated with substantial economic losses [18].

**Figure 1:**
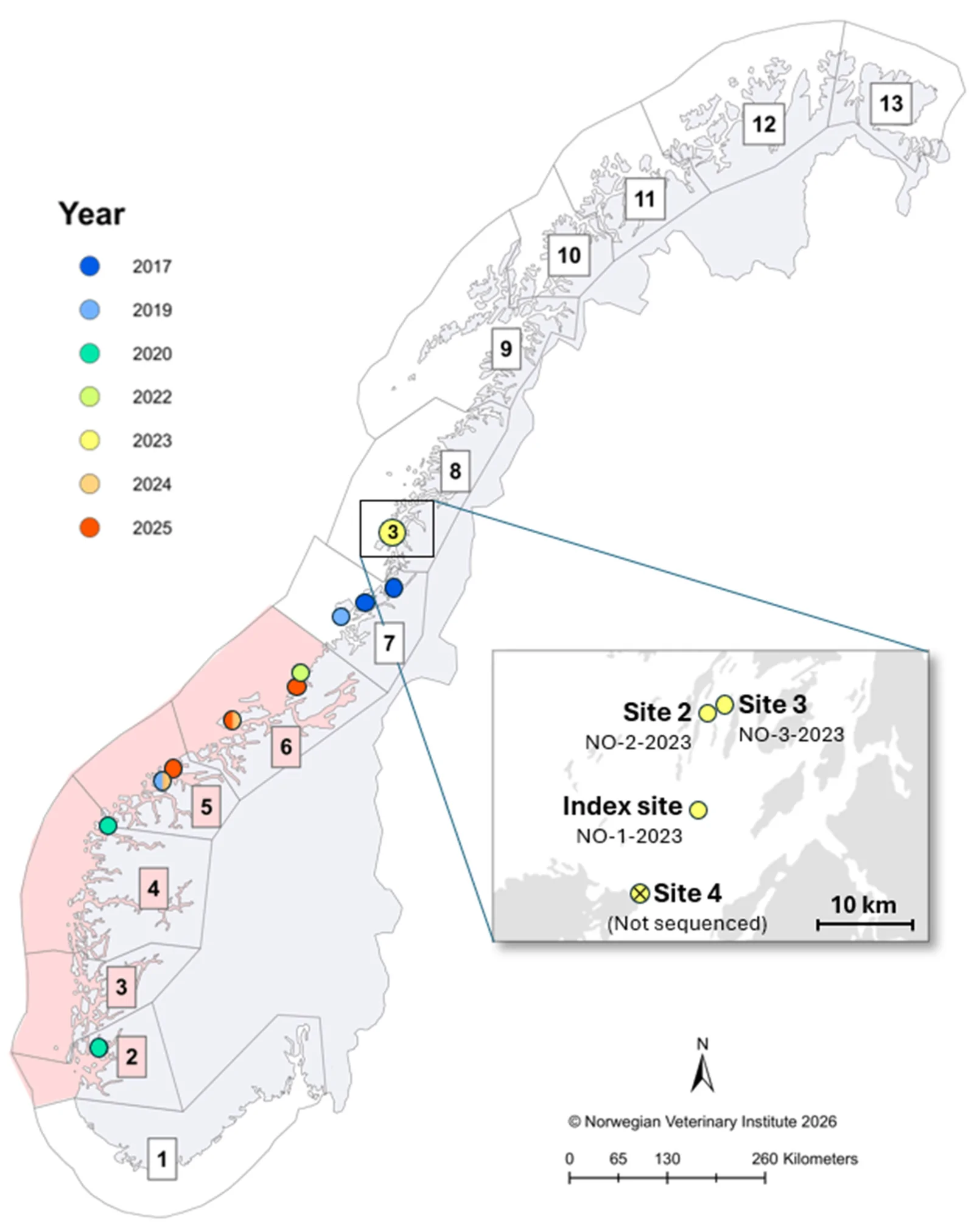
Geographic distribution of Norwegian aquaculture production areas (PAs) and SAV2-positive sampling sites included in this study. Norwegian aquaculture production areas (PA1–PA13) are shown from south to north, with the PD zone highlighted in red (PA2–PA6). Sampling locations represent Atlantic salmon (*Salmo salar*) sites from which SAV2 whole-genome sequences were generated between 2017 and 2025. Points indicate the approximate geographic origin of each sequenced virus and are coloured according to sampling year. Sites sampled in multiple years are shown using split colours. The inset shows a zoomed view of four neighbouring aquaculture sites in PA8 associated with SAV2 detections in autumn 2023. Sample IDs NO-1-2023, NO-2-2023, and NO-3-2023 denote the sequenced SAV2-positive samples included in this study, while Site 4 represents an SAV2-positive site from which no whole-genome sequence was obtained. Map source: Norwegian Veterinary Institute.

Norwegian salmonid aquaculture is organized into geographically defined production areas (PAs) (Figure 1) [19]. Ongoing epidemics caused by both SAV2 and SAV3 have been well documented [7,20]. The Norwegian PD regulation defines the PD zone as the area spanning from PA2 to PA6 and the areas north and south of this zone are considered surveillance zones, where the objective is to prevent PD from being established (Figure 1) [21]. According to the regulation, vessels that move out of the PD zone after transporting live fish must complete cleaning, disinfection and a quarantine time before entering a surveillance zone [21]. PD has occurred endemically in the PD zone, but there has been a decline in the number of cases in recent years and there was a decrease in the number of PD cases from 98 cases in 2022 to 58 cases in 2023 [22].

The distribution of the two genotypes shows a clear geographical pattern: outbreaks caused by SAV3 are mainly detected in PA3 and PA4, covering Vestland county. SAV3 was also detected in PA2 (county Rogaland) and PA5 (county Møre og Romsdal) for many years. However, as of 30 April 2026, no new cases of PD have been detected in PA2 since 2022, while no new cases attributed to SAV3 have been detected in PA5 since 2023 [11,23]. SAV2 has historically been confined to PA5–PA7, spanning county Møre og Romsdal to the northern border of county Trøndelag [11], with sporadic cases detected outside of the endemic areas PA5 and PA6. This relatively stable distribution has formed the basis for surveillance and control strategies aimed at limiting the further spread of pancreas disease within the Norwegian aquaculture industry.

Although anthropogenic factors, particularly the movement of live fish between production sites, are considered major drivers of long-distance transmission of SAV [24], natural reservoirs and environmental persistence may also contribute to virus epidemiology [18,25,26,27,28]. Distinguishing between recent introductions and local persistence is therefore important for understanding transmission dynamics and for informing disease control measures.

The application of whole-genome sequencing has substantially improved the resolution of molecular epidemiological investigations of aquatic viral diseases. SAV1 and SAV6 were first sequenced using nanopore technology in 2018 [29], and subsequent studies have demonstrated the value of whole-genome data for reconstructing transmission pathways and investigating the epidemic spread of SAV [24]. Previous phylogenetic studies have further shown that Norwegian SAV2 viruses originate from a Scottish ancestor and have diversified into two major lineages, designated SAV2a and SAV2b [30].

In 2023, SAV2 was detected within the surveillance zone at four Atlantic salmon aquaculture sites along the Helgeland coast in PA8 (Figure 1), north of the previously recognized distribution of the virus [22]. These detections provided a unique opportunity to investigate the spread of SAV2 in Norwegian aquaculture. The aim of the present study was to combine epidemiological information and whole-genome sequencing data to investigate the origin of the SAV2 viruses detected in Helgeland and to assess whether the outbreaks were consistent with local persistence or recent introduction from established SAV2-endemic regions further south.

## 2. Materials and Methods

### 2.1. Sample Collection and Nucleic Acid Extraction

Heart and kidney tissues were collected by fish health personnel from Atlantic salmon (*Salmo salar*) naturally infected with SAV2. Samples were submitted to the Norwegian Veterinary Institute (Ås, Norway) for routine diagnostics, and samples from 2017 to 2025 were included in the study based on spread in space and time, aiming to include samples from different geographical clusters (Table 1). Intra-fish pools of heart and kidney tissues from 15 fish originating from 13 sites, including three of the four sites in PA8, were homogenized in 600 μL MagNA Pure Tissue Lysis Buffer (Roche, Basel, Switzerland) using a 3 mm tungsten bead and a TissueLyser II instrument (Eppendorf, Hamburg, Germany) at 25 Hz for 10 min. Nucleic acids were extracted from 200 μL of homogenate using a MagNA Pure 24 Instrument (Roche) with the Pathogen 200 protocol and Whole Blood sample setting. Extracted nucleic acids were eluted in 50 μL MagNA Pure Elution Buffer (Roche) and quantified using a Multiskan Sky spectrophotometer (Thermo Fisher Scientific, Waltham, MA, USA).

### 2.2. cDNA Synthesis and PCR Amplification

Between 100 and 400 ng of extracted nucleic acid was used for cDNA synthesis with ProtoScript^®^ II Reverse Transcriptase (New England Biolabs, NEB, Ipswich, MA, USA). Reverse transcription was performed at 42 °C for 60 min, followed by enzyme inactivation at 80 °C for 5 min. PCR amplification was performed using LongAmp^®^ Taq 2× Master Mix (NEB). Each reaction contained 1 μL cDNA template and 0.5 μM of each primer in a final volume of 25 μL. Primer sequences are listed in Table S1. Cycling conditions consisted of an initial denaturation at 94 °C for 30 s, followed by 35 cycles of 94 °C for 30 s, 56 °C for 1 min and 65 °C for 4 min, with a final extension at 65 °C for 10 min. Amplification products were verified by electrophoresis on 1% agarose gels. For each fish, equal volumes (10 μL) of the three amplicons were pooled and purified using AMPure XP beads (Beckman Coulter, Brea, CA, USA) at a 0.5× bead-to-sample ratio. Purified DNA was eluted in 21 μL nuclease-free water and quantified using a NanoDrop spectrophotometer (Thermo Fisher Scientific). Amplicon pools were normalized to 36.4 ng/μL prior to library preparation.

### 2.3. Library Preparation and Nanopore Sequencing

Sequencing libraries were prepared using Oxford Nanopore Technologies (ONT, Oxford, UK) ligation-based protocols. Purified amplicon pools (400 ng) were end-repaired and dA-tailed using the NEBNext^®^ Ultra™ II End Repair/dA-Tailing Module (NEB) according to the manufacturer’s instructions. Samples were barcoded using the Native Barcoding Kit 24 V14 (SQK-NBD114.24; ONT) and pooled in equimolar amounts. Following purification with AMPure XP beads, sequencing adapters were ligated using Quick DNA Ligase (NEB). The final library was purified according to ONT recommendations and loaded onto an R10.4.1 MinION flow cell (ONT).

### 2.4. Sequence Processing and Consensus Generation

Basecalling, demultiplexing, quality control, and read filtering were performed using the Nepal pipeline [31], incorporating Dorado v1.3.0, Nanoplot v1.40.2, PycoQC v2.5.2, and Nanofilt v2.8.0. Reads were filtered using a minimum quality score of 9, retaining reads between 3000 and 10,000 bp in length. The bioinformatics workflow is shown in Figure S1. Filtered reads were mapped against the SAV2 reference genome (OL754582) using Geneious Prime v2025.1.1. Only samples yielding sufficient sequencing data (minimum sequencing depth >400) to cover the near-complete SAV2 genome (>10 kb) were carried forward for analysis (Table 1), except for sample MR-1-2019, which had a minimum sequencing depth of 2 (Table 1; Figure S2). Consensus sequences were generated following manual inspection of alignments, including verification of putative insertion/deletion events and potential frameshift mutations. Primer sequences were removed prior to consensus generation. Consensus genome sequences generated in the present study were deposited in GenBank under accession numbers PX864625–PX864639.

### 2.5. Phylogenetic Analysis

**Figure 2:**
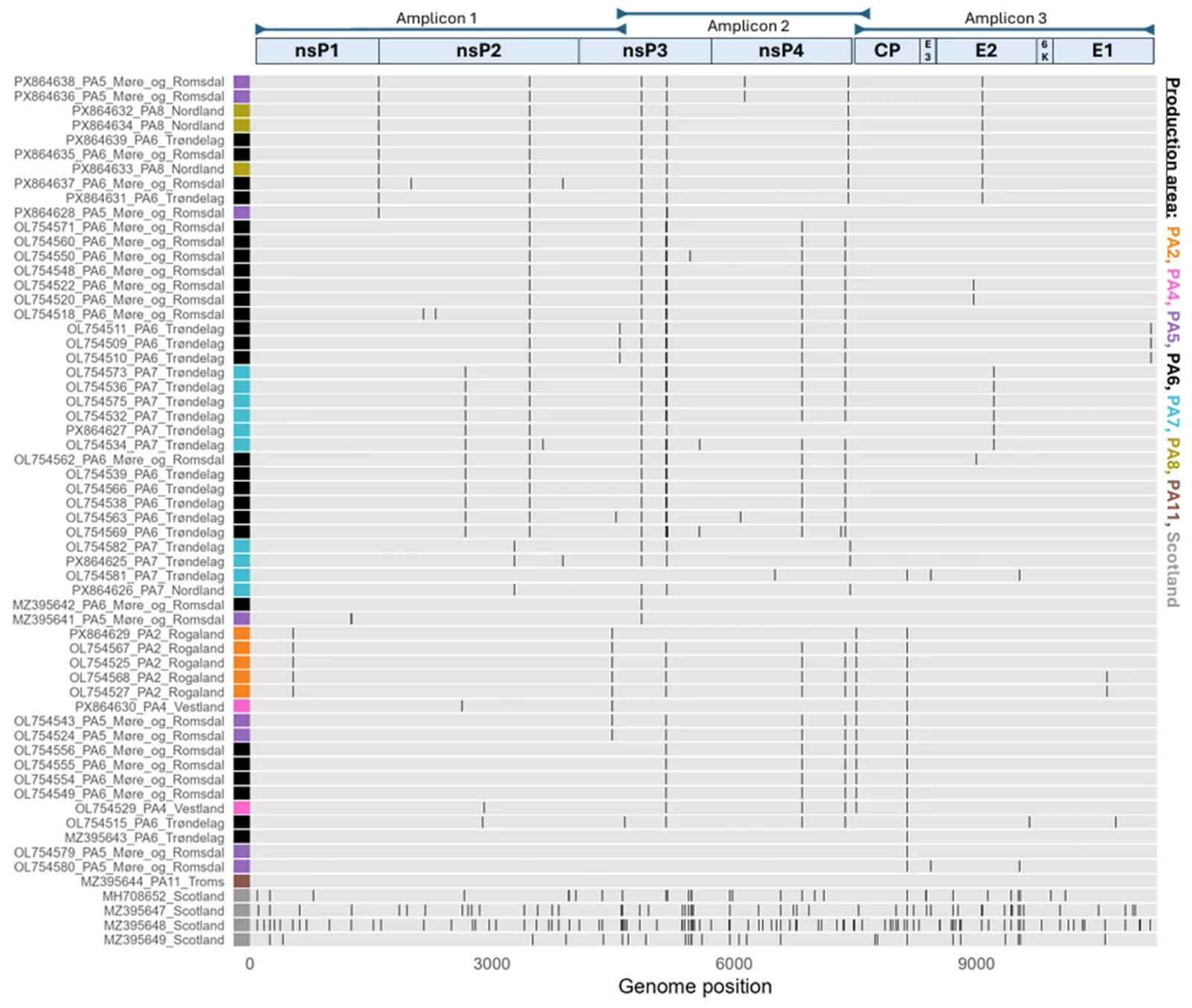
Genome-wide nucleotide variation among SAV2 isolates generated in this study (accession numbers prefixed with PX) and previously published SAV2 reference genomes. Complete SAV2 genome sequences were aligned against the SAV2 genome sequence MZ395644. Each row represents a single genome, and vertical black bars indicate nucleotide positions that differ from the reference sequence MZ395644. The alignment was used for phylogenetic inference shown in Figure 3. The x-axis indicates nucleotide position along the SAV2 genome. The 24-bp region within the nsP3 coding sequence containing a known deletion is also excluded in this alignment. The approximate positions of the PCR amplicons generated by the primers listed in Table S1 are indicated at the top of the figure.

Consensus sequences generated in the present study were analyzed together with complete SAV2 genome sequences (>10 kb) published previously [30,32]. Sequences were aligned against SAV2 genome sequence MZ395644 (Figure 2). A short region within the nsP3 coding sequence containing a known 24 bp deletion was excluded from subsequent analyses (Figure S4). Time-scaled phylogenetic inference was performed in BEAST v2.7.7 using an uncorrelated lognormal relaxed molecular clock. Sequence evolution was modelled using the TN+F+I substitution model, which was identified as the best-fitting model by ModelFinder implemented in IQ-TREE v3.1.2. A coalescent constant population prior was used, assuming a constant effective population size through time. Markov chain Monte Carlo (MCMC) analyses were run for 100 million generations, with parameters and trees sampled every 10,000 generations. Convergence and mixing were assessed in Tracer. All key parameters achieved effective sample size (ESS) values greater than 600, indicating adequate sampling of the posterior distribution and satisfactory convergence of the MCMC chains. Following MCMC analysis, a maximum clade credibility (MCC) tree was generated in TreeAnnotator v2.7.7 after discarding the first 20% of sampled trees as burn-in. Node heights were summarized using the common ancestor (CA) method. Node ages, posterior probabilities, and 95% highest posterior density (HPD) intervals were inspected and extracted using FigTree v1.4.4.

**Figure 3:**
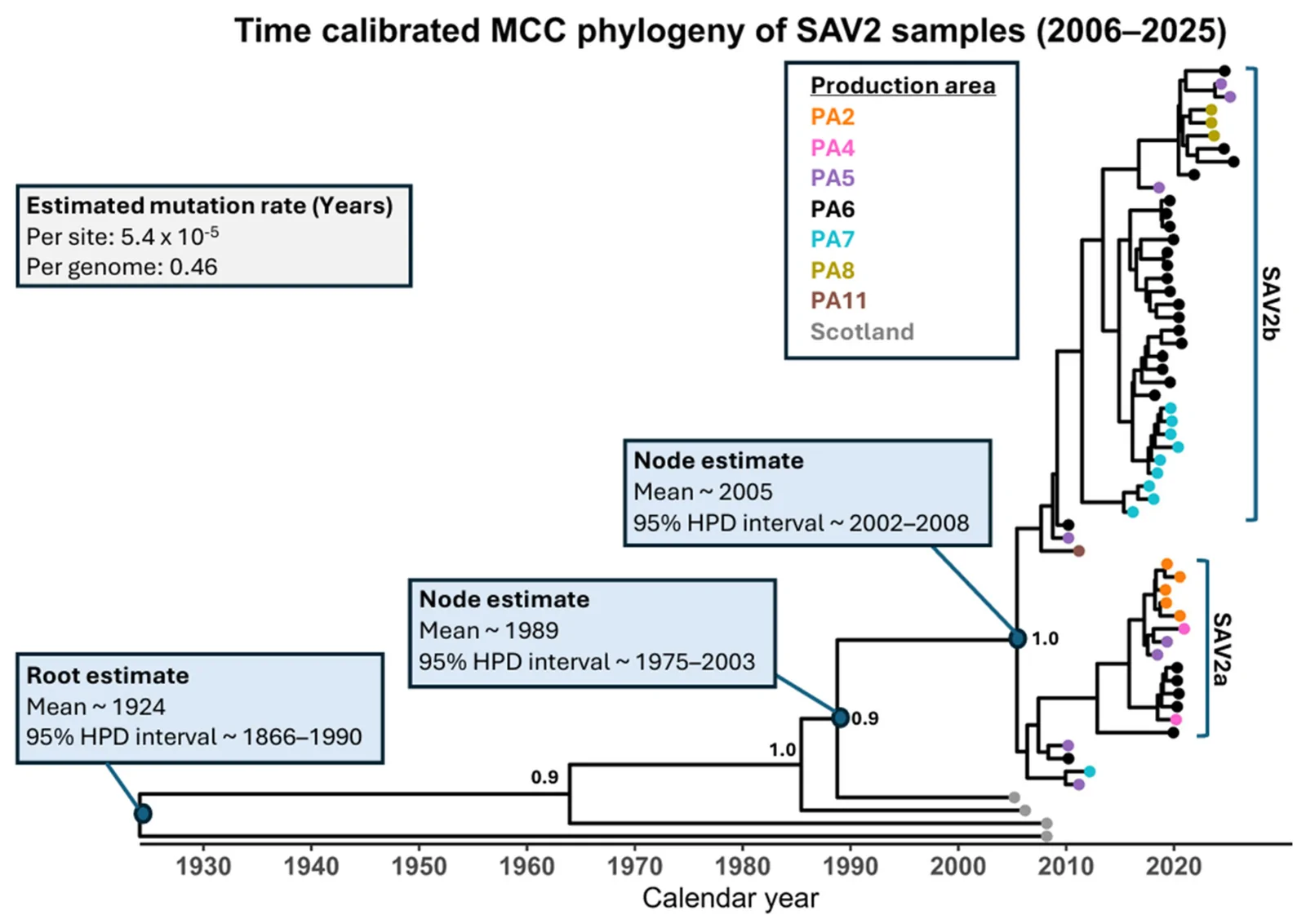
Time-calibrated maximum clade credibility (MCC) phylogeny of SAV2 sequences collected between 2006 and 2025. The phylogeny was inferred using a Bayesian Markov chain Monte Carlo (MCMC) approach implemented in BEAST and summarized as a maximum clade credibility (MCC) tree. Branch lengths are scaled in calendar years, with the x-axis representing estimated divergence times. Colored tip symbols indicate phylogenetic assignment to the respective PA groups (PA2, PA4, PA5, PA6, PA7, PA8, and PA11), while grey symbols represent sequences originating from Scotland. Blue nodes indicate selected ancestral node estimates. Text boxes show the estimated ages of key ancestral nodes, including the posterior mean date and the 95% highest posterior density (HPD) interval. The inferred substitution rate was 5.4 × 10^−5^ substitutions per site per year, corresponding to approximately 0.65 substitutions per genome per year. The most recent common ancestor (MRCA) of the analyzed SAV2 dataset was estimated to have existed around 1924 (95% HPD: 1866–1990). The closest Scottish relative of the Norwegian isolates was estimated to have diverged around 1989 (95% HPD: 1975–2003), while the common ancestor of the two major Norwegian clades was estimated to have existed around 2005 (95% HPD: 2002–2008).

## 3. Results and Discussion

### 3.1. Description of Outbreak and Protective Measures

On 19 September 2023, a PD suspicion (SAV2) outside the PD zone arose at a salmon grow out sea site, the index site, in Helgeland (PA8), with a subsequent PD diagnosis three days later (Figure 1). Consequently, a restricted zone was established by The Norwegian Food Safety Authority consisting of a protection zone surrounded by a surveillance zone [33]. This was the first PD outbreak outside the PD zone since 2019. The index site was depopulated of all fish by 8 October following an order from the Norwegian Food Safety Authority. Three additional outbreaks occurred at salmon grow out sites in PA8, within the protection zone, in the latter half of November. These sites were depopulated during mid-December, with fish either slaughtered or discarded. Samples from the index site, Site 2, and Site 3, with sample IDs NO-1-2023, NO-2-2023, and NO-3-2023, respectively, were whole-genome sequenced in this study (Figure 1). A sample from Site 4 was also subjected to whole-genome sequencing; however, the sequencing data obtained were of insufficient quality for downstream analysis.

### 3.2. Epidemiological Investigation

The Norwegian Veterinary Institute carried out an epidemiological investigation in collaboration with the Norwegian Food Safety Authority based on data provided by the companies running the four affected sites, including information on the origin of the fish, fish movement and information about vessels operating in the area. The fish originated from four different hatcheries, all located outside the PD zone. The index site was stocked from September to November in 2022. Site 4, located southwest of the index site, was stocked from August to November 2022, while Site 2, one of the two northeastern sites, was stocked in late April and early May 2023 (Figure 1, Table 2). The population at Site 2 was subsequently split, with some of the fish being transferred to the neighboring Site 3 in October 2023. PD was suspected and eventually confirmed at Sites 2 and 3 in November 2023 and at Site 4 in December 2023. The fish at the index site were deloused for the very first time in August 2023, one month prior to the initial PD suspicion. As the delousing vessel was moved directly from the index site to Site 4, vessel traffic may have contributed to the spread of the virus. However, based on the geographical proximity of the sites in PA8, horizontal transmission of infection is a possible explanation for the spread of infection to all three secondary sites within the area. This is also supported by the initial epidemiological assessment of the area following the initial outbreak. How SAV2 was introduced to the area remains unknown [22].

### 3.3. Nanopore Sequencing of SAV from Norwegian Field Samples

While the epidemiological investigation identified potential transmission pathways between affected sites, the source of SAV2 introduction remained unresolved. To provide additional insight into the origin and spread of the virus, nanopore sequencing was used to generate SAV genomes for comparative phylogenetic analysis. Nanopore sequencing generated high-quality SAV genomic data from Atlantic salmon sampled across multiple Norwegian production areas (Table 1; Figure 1). Three PCR amplicons spanning the SAV genome (Figure 2) were successfully sequenced from heart and kidney tissues of 15 individuals using the ONT MinION platform. After 24 h, the sequencing run produced 3.17 million reads, of which 2.55 million passed quality filtering (Q > 9), with an N50 read length of 1897 bp. A total of 94.6% of reads were successfully demultiplexed, ensuring reliable barcode assignment for all samples. On average, approximately 10.6 kb of the SAV2 genome was reconstructed from 15 individuals, corresponding to ~90% genome coverage. Amplicons 1 and 3 consistently produced the highest sequencing depth and recovery rates across samples, whereas Amplicon 2 showed reduced amplification efficiency and lower sequence coverage. Fourteen samples achieved a minimum sequencing depth >400 across the recovered genome, whereas sample PX864628 represented a notable outlier, with a minimum sequencing depth of 2. Despite its low and uneven coverage, manual inspection of read alignments revealed a coherent read pattern with limited conflicting base calls and no evidence of systemic sequencing artefacts (Figure S2). Furthermore, the consensus sequence generated from the recovered genomic region was highly consistent with an SAV2 genome obtained from the same location approximately five years later, supporting its biological validity. Consequently, PX864628 was retained for downstream analyses, although regions with very low coverage should be interpreted with caution. Overall, sufficient genomic data was obtained from all 15 samples to support downstream evolutionary analyses (Table 1).

### 3.4. Genetic Diversity of Norwegian SAV2

**Figure 4:**
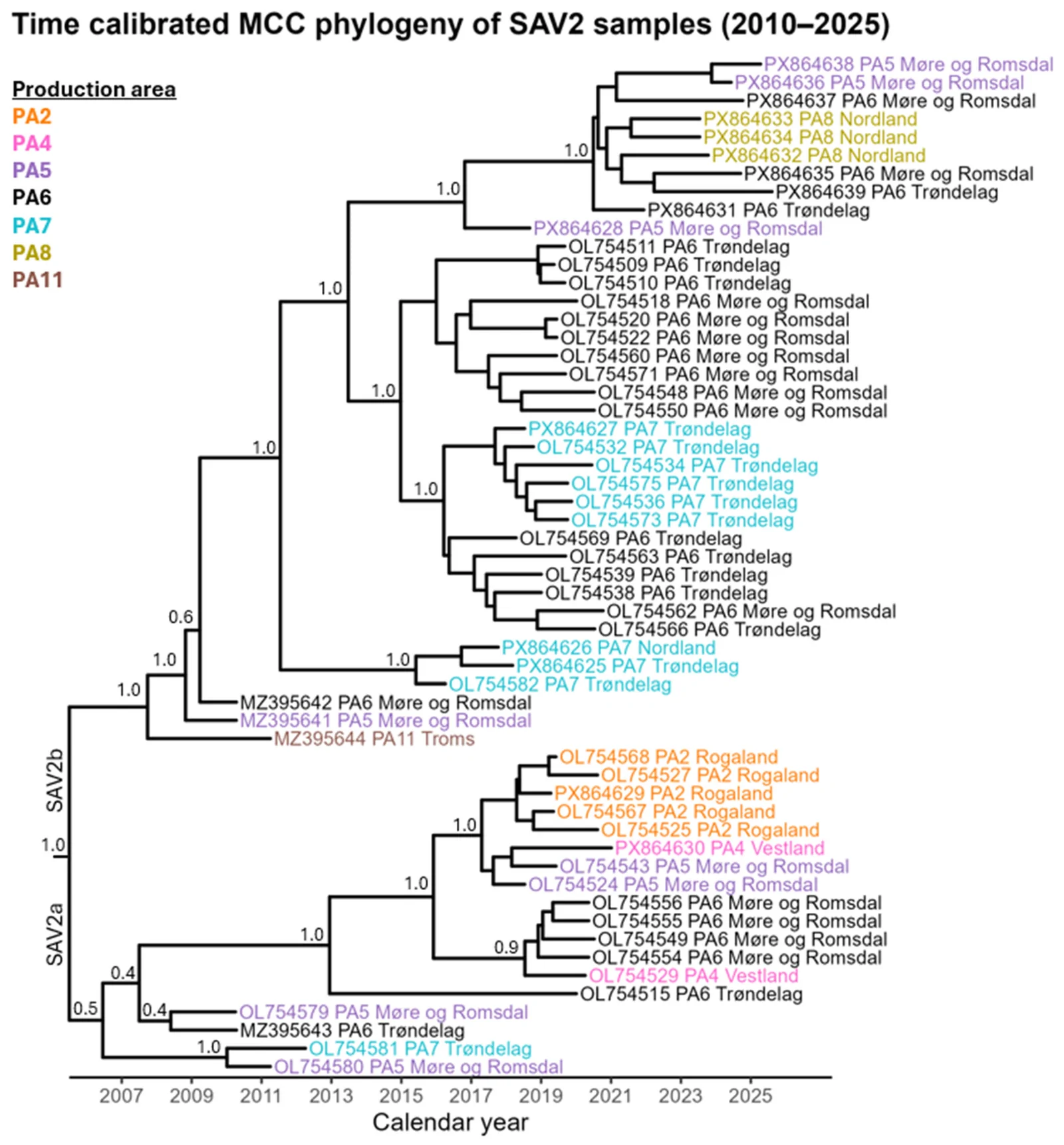
Time-calibrated maximum clade credibility (MCC) phylogeny of SAV2 genomes collected from Norwegian Atlantic salmon production areas between 2010 and 2025. Bayesian time-scaled phylogenetic inference was performed in BEAST v2.7.7 using an uncorrelated lognormal relaxed molecular clock, the TN+F+I substitution model with gamma-distributed rate heterogeneity among sites, and a coalescent constant population prior. The analysis included complete SAV2 genome sequences (>11 kb) generated in the present study together with previously published SAV2 genomes. Sequences were aligned to the SAV2 reference genome MZ395644, and a 24-bp region within the nsP3 coding sequence containing a known deletion was excluded from the analysis. Branch lengths are proportional to time, with the x-axis indicating calendar year. Tip labels are colored according to production area, as shown in the legend. The phylogeny resolves the two major SAV2 lineages, SAV2a and SAV2b. Numbers at select internal nodes indicate posterior clade probabilities. Sequences generated in this study have GenBank accession numbers PX864625–PX864639.

The SAV2 genomes generated in this study were analyzed together with previously published SAV2 genomes (Figure 2, Figure 3 and Figure 4). Multiple sequence alignment of Norwegian and Scottish SAV2 genome sequences (Figure 2) identified a 24-bp deletion previously noted by Hjortaas et al. [32] (Figure S3). Although the deletion was observed in genome sequences published by Macqueen et al. [30], none of the SAV2 sequences generated in the present study contained it, including strains collected in close temporal and geographical proximity to those viruses. As we could not determine whether the deletion represented a genuine genomic feature of SAV2 or originated from subgenomic RNA, the affected region was excluded from downstream phylogenetic analyses. Multiple sequence alignment further revealed complete nucleotide identity between SAV2 strains from Helgeland sites in PA8 and viruses from three separate sites in PA6 sampled in 2022, 2024, and 2025 (Figure 2). Analysis of the translated protein sequences from the aligned genomes within the SAV2b clade identified only four amino acid substitutions, observed on more than one occasion, among SAV2 genomes sampled between 2016 and 2025, indicating a high degree of protein sequence conservation over time. The substitutions comprised two changes within the nsP4 region (N → D and P → A) and two within the E2 protein (A → E and I → T) (Figure S4).

### 3.5. Phylogenetic Analysis of Norwegian SAV2

Time-scaled phylogenetic analysis of Norwegian and Scottish SAV2 genomes revealed the two major Norwegian lineages, designated SAV2a and SAV2b, coined by Macqueen et al. [30] (Figure 3 and Figure 4). The most recent common ancestor of the Norwegian SAV2 population was estimated to have existed around 2005 (95% HPD: 2002–2008) (Figure 3), suggesting that the currently circulating Norwegian SAV2 viruses originated from a relatively recent ancestral population introduced between 2005 and the first reported case in 2010 [20]. In contrast, Scottish SAV2 sequences occupied more basal positions in the phylogeny and displayed greater genetic diversity, consistent with a longer evolutionary history of SAV2 in Scotland than in Norway [20].

The phylogenetic structure, together with the high nucleotide similarity observed among Norwegian isolates, supports the hypothesis that SAV2 was introduced into Norway on a single occasion between 2005–2010 [32], followed by local dissemination and diversification into the SAV2a and SAV2b lineages [30]. This interpretation is further supported by the remarkably limited genetic variation observed across the dataset (Figure 2). Multiple sequence alignment identified complete nucleotide identity among viruses collected from different production areas and across multiple years (Figure 2), and only a small number of nonsynonymous substitutions were detected among genomes sampled between 2016 and 2025 (Figure S4). Such limited sequence diversity is consistent with a relatively recent common ancestry and subsequent spread within the Norwegian production system.

Although the two Norwegian SAV2 lineages, SAV2a and SAV2b, are clearly distinguishable, their geographical distributions indicate substantial movement of SAV2 between production areas. SAV2 is primarily associated with its endemic zone in production areas PA5 and PA6 [11]. SAV2b has previously been reported from production areas located south of the endemic SAV2 zone [30]. Phylogenetic analyses suggest that the SAV2a viruses detected in PA2, in 2019, likely originated from PA5, whereas the SAV2a viruses detected in PA4 in 2020 likely originated from PA6. The genome sequence generated in the present study reveals an additional introduction event into northern PA4 in 2021, clustering with SAV2a viruses from PA5 and indicating a separate introduction from this production area (Figure 4). Together, these findings suggest that SAV2 has been introduced into PA4 from the endemic area on at least two independent occasions.

Northward spread of SAV2b beyond the restriction zone was previously documented in PA7 in 2017 [30] and is now also evident in PA8 based on sequences obtained in 2023 (Figure 4). Sequences from Helgeland in PA8 clustered tightly with viruses sampled from multiple locations in PA6, including isolates collected in 2022, 2024, and 2025. Furthermore, complete nucleotide identity was observed between the PA8 and PA6 viral genomes. Together, these findings indicate a recent epidemiological connection between the two production areas and suggest that SAV2 was likely introduced into PA8 from PA6. While phylogenetic data alone cannot determine the precise transmission route, the close genetic relationship and absence of substantial sequence divergence support a relatively recent transfer event. Although re-introduction from unsampled reservoirs, including marine fauna, cannot be excluded, the close genetic relationship between the PA6 and PA8 genomes suggests direct epidemiological linkage between the two production areas and is compatible with transmission through human-mediated movement related to aquaculture production. Taken together, these findings demonstrate that the SAV2 outbreaks detected on the Helgeland coast during 2023 originated from the established Norwegian SAV2b lineage and most likely represent recent northward spread from PA6. This highlights the continued importance of inter-regional transmission pathways in shaping the epidemiology of pancreas disease in Norwegian aquaculture.

## Figures and Tables

**Table 1:** Sequencing depth statistics for SAV2 genomes generated in this study. Mean, minimum and maximum sequencing depths were calculated from mapped sequence reads across each SAV2 genome. Genomes were recovered from pooled heart and kidney tissue of Atlantic salmon (*Salmo salar*) sampled in Norwegian production areas (PA) between 2017 and 2025. Sample IDs are prefixed with county codes as follows: TR = Trøndelag, NO = Nordland, MR = Møre og Romsdal, RO = Rogaland, and VL = Vestland. GenBank accession numbers are provided for all sequences included in the phylogenetic and comparative genomic analyses.

Sample Information				Sequencing Depth (Reads)		
Sample ID	GenBank Accession	Sampling MM.YYYY	Production Area (PA)	Mean Depth	Minimum Depth	Maximum Depth
TR-1-2017	PX864625	05.2017	PA7	64,034	23,381	127,241
NO-1-2017	PX864626	07.2017	PA7	18,686	6941	41,533
TR-1-2019	PX864627	09.2019	PA7	3269	972	6241
MR-1-2019	PX864628	02.2019	PA5	17,444	2 *	30,336
RO-1-2020	PX864629	08.2020	PA2	14,789	10,877	28,070
VL-1-2020	PX864630	01.2020	PA4	1766	719	4066
TR-1-2022	PX864631	05.2022	PA6	28,213	10,499	45,652
NO-1-2023	PX864632	09.2023	PA8	5104	425	10,368
NO-2-2023	PX864633	11.2023	PA8	12,437	4003	28,299
NO-3-2023	PX864634	11.2023	PA8	7192	2887	15,473
MR-1-2024	PX864635	12.2024	PA6	13,003	1955	25,092
MR-2-2024	PX864636	05.2024	PA5	10,934	1601	21,674
MR-1-2025	PX864637	05.2025	PA6	15,333	3357	28,516
MR-2-2025	PX864638	06.2025	PA5	21,825	1046	46,835
TR-1-2025	PX864639	06.2025	PA6	4105	475	7235

* This sample yielded poor sequencing depth in the nsP1–nsP3 amplicon. Genome sequence was included in this study after manual inspection of sequence alignment (Figure S2).

**Table 2:** Overview of fish movements, stocking history, and official PD suspicion and confirmation events at sites in PA8 during autumn and winter 2023. Dates are in the format MM.YYYY.

Site	Stocking Period	Source of Stocked Fish	Date of PD Suspicion	Date of PD Confirmation	Site Emptied
1 (Index)	09–11.2022	Hatcheries outside PD zone	09.2023	09.2023	10.2023
2	04–05.2023	Hatchery outside PD zone	11.2023	11.2023	12.2023
3	10.2023	Site 2	11.2023	11.2023	12.2023
4	08–11.2022	Hatchery outside PD zone	11.2023	12.2023	12.2023

## Data Availability

Consensus genome sequences generated in the present study were deposited in GenBank under accession numbers PX864625-PX864639.

## Supplementary Materials

The following supporting information can be downloaded at: https://www.mdpi.com/article/10.3390/v18101089/s1. Figure S1: Bioinformatics workflow for Nanopore sequence analysis and phylogenetic reconstruction. Raw sequencing data were generated using Oxford Nanopore sequencing and acquired with MinKNOW, producing .POD5 files. Basecalling, demultiplexing, quality control, and read filtering were performed using the Nepal pipeline, incorporating Dorado v1.3.0, Nanoplot v1.40.2, PycoQC v2.5.2, and Nanofilt v2.8.0, resulting in filtered .FASTQ files. Consensus sequences were assembled in Geneious v2025.1.1 and exported as .FASTA files. Consensus sequences were subsequently aligned to the reference SAV2 sequence in Geneious v2025.1.1 to generate an aligned .FASTA dataset. ModelFinder v3.1.2 was used to find best fitting substitution model. BEAUti v2.7.8 was used to prepare the Bayesian phylogenetic analysis input file (.XML), which was analyzed in BEAST v2.7.7 to infer a time-calibrated phylogeny, producing .trees and log (.log) files. Convergence and parameter estimates were assessed in Tracer v1.7.2. The posterior tree distribution was summarized using TreeAnnotator v2.7.7 to generate a maximum clade credibility tree (.tree). Finally, the annotated phylogenetic tree was visualized and plotted in RStudio v2025.09.2 and exported as a .PNG image; Figure S2: Read alignment, coverage, and consensus generation for the SAV2 genome sequence PX864628, representing the dataset with the lowest sequencing depth among all samples analysed. Sequencing reads from the first amplicon spanning the 5′ region of the non-structural polyprotein (nsP1–nsP3) were mapped to the SAV2 reference genome. The upper panels show the consensus sequence, coverage profile, and nucleotide identity, while the lower panel displays the individual forward and reverse reads used for consensus generation. Disagreements with consensus sequence are highlighted on aligned sequences. Although PX864628 exhibited the poorest overall sequencing performance (mean coverage 17 444, minimum coverage 2, maximum coverage 30 336), the alignment across the first amplicon showed a coherent read pattern with limited conflicting base calls and no evidence of systematic misalignment or clustered sequencing artefacts. Manual inspection of the alignment supported the consensus sequence generated for this region. Importantly, the resulting consensus was highly consistent with the corresponding genomic region of the PX864636 SAV2 sequence recovered from the same location approximately five years later, providing independent biological support for the sequence reconstruction. Together, the read-level concordance and agreement with an epidemiologically related strain indicate that the consensus sequence for the first amplicon is likely reliable despite the low and uneven sequencing depth. Regions with very low coverage should nevertheless be interpreted with caution; Figure S3: Alignment of the nsP3 genomic region containing a 24-nucleotide deletion in SAV2 genomes. Nucleotide alignment of SAV2 genomes spanning positions 5241–5311 relative to the reference genome MZ395648. Each row represents a single isolate and colored nucleotides indicate the aligned sequence. Gaps (–) denote the 24-nt deletion located within the nsP3 coding region. Viral samples are colored according to production area: PA2 (orange), PA4 (pink), PA5 (purple), PA6 (blue), PA7 (cyan), PA8 (green), and PA11 (brown); Scottish isolates are shown in black. The deletion is present in the majority of Norwegian SAV2 genomes across multiple production areas and years, while several Norwegian isolates and all Scottish reference genomes retain the full-length sequence. This deletion region was excluded from the Bayesian phylogenetic analyses to avoid artefactual clustering driven by the indel rather than nucleotide substitutions. The alignment illustrates the distribution of the deletion among Norwegian SAV2 lineages; Figure S4: Amino acid variation across translated SAV2 polyproteins. Complete SAV2 genome sequences were translated and aligned relative to the reference genome MZ395644. Each row represents a single SAV2 isolate, and vertical black bars indicate amino acid positions that differ from the reference sequence. Colored bars adjacent to isolate names denote production areas as defined in legend. Protein boundaries are shown above the alignment, including the non-structural proteins (nsP1–nsP4) and structural proteins (capsid protein [CP], E3, E2, 6K, and E1). Most amino acid substitutions were observed within nsP3, whereas relatively few non-synonymous changes occurred in the remaining genomic regions. Scottish isolates (MH708652, MZ395647, MZ395648, and MZ395649), which were excluded from the Bayesian phylogenetic analysis presented in Figure 3, are included to illustrate the greater amino acid diversity observed among Scottish SAV2 genomes compared with contemporary Norwegian isolates. For simplicity, amino acids resulting from unambiguous nucleotides at position 5250 (Figure S3) are not indicated by black bars. Protein positions are shown on the x-axis; Table S1: PCR primers used for amplification of SAV2 genomic regions for whole-genome sequencing. Primer pairs targeting structural and non-structural regions of the SAV2 genome were used to generate overlapping amplicons spanning the viral genome. Primers for amplification of ORF2 and the nsP1–nsP3 region were adapted from Gallagher et al. [29], whereas the nsP2–ORF2 primer pair was designed in the present study based on the SAV2 genome sequence and modified from primers described by Tighe et al. [9].

## Abbreviations

The following abbreviations are used in this manuscript:

SAV	Salmonid alphavirus
PD	Pancreas disease
SPDV	Salmon pancreas disease virus
SDV	Sleeping disease virus
WGS	Whole-genome sequencing
PA	Production area
MCMC	Markov chain Monte Carlo
MCC	Maximum clade credibility
HPD	Highest posterior density
MRCA	Most recent common ancestor
