# Supplementary material for: Molecular Epidemiological Investigation of Salmonid Alphavirus Genotype 2 (SAV2) Emergence on the Helgeland Coast of Norway in 2023
Source: Viruses. 2026 Oct 2;18(10):1089. doi: 10.3390/v18101089 (PMC13709673; doi:10.3390/v18101089)
Supplement: Supplementary file 1 [file viruses-18-01089-s001.zip › viruses-4571795-supplementary.pdf]

Supplementary material

Table S1. PCR primers used for amplification of SAV2 genomic regions for whole-genome sequencing. Primer pairs targeting structural and non-structural regions of the SAV2 genome were used to generate overlapping amplicons spanning the viral genome. Primers for amplification of ORF2 and the nsP1–nsP3 region were adapted from Gallagher (2018), whereas the nsP2–ORF2 primer pair was designed in the present study based on the SAV2 genome sequence and modified from primers described by Tighe (2020).

Amplicon	Primer	Sekvens (5′ → 3′)	Citation
ORF2	Structural-F	CMAACTCAGCCTAYCGCCAG	[24]
	Structural-R	GCACTTCTTCACCACGCAG	
nsP1-3	Non-structural/a-F	AGACTGCGTTTCCAGGGTT	[24]
	Non-structural/a-R	ATGTCGGTCAGTTGAGGGC	
nsP2-ORF2	Non-structural/b2-F	TTTCAAGTCCGTCTCGGTCG	This study
	Non-structural/b2-R	YTTGGGTTTTTCTCCGTTGG	[9]

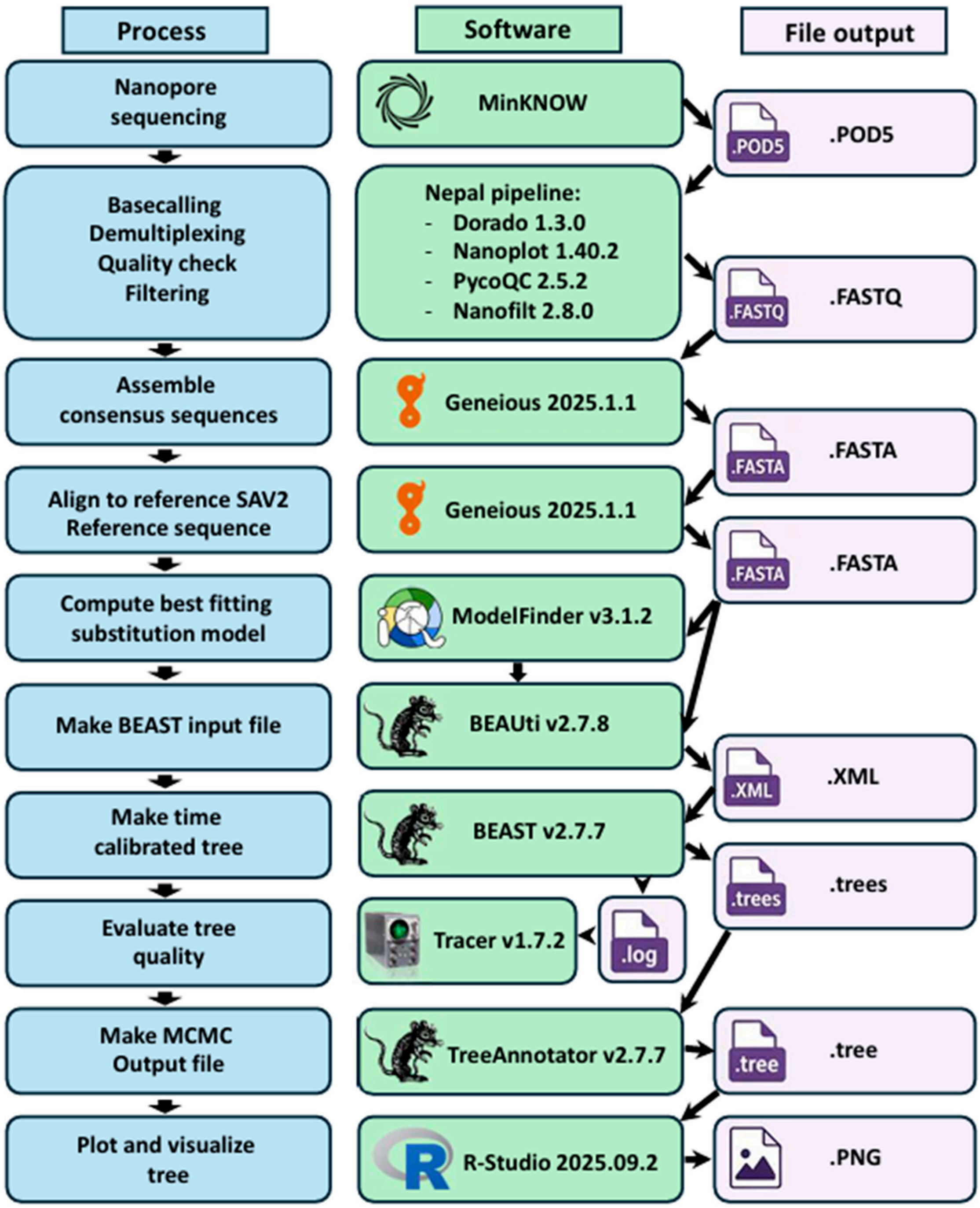

Figure S1. Bioinformatics workflow for Nanopore sequence analysis and phylogenetic reconstruction. Raw sequencing data were generated using Oxford Nanopore sequencing and acquired with MinKNOW, producing .POD5 files. Basecalling, demultiplexing, quality control, and read filtering were performed using the Nepal pipeline, incorporating Dorado v1.3.0, Nanoplot v1.40.2, PycoQC v2.5.2, and Nanofilt v2.8.0, resulting in filtered .FASTQ files. Consensus sequences were assembled in Geneious v2025.1.1 and exported as .FASTA files. Consensus sequences were subsequently aligned to the reference SAV2 sequence in Geneious v2025.1.1 to generate an aligned .FASTA dataset. ModelFinder v3.1.2 was used to find best fitting substitution model. BEAUti v2.7.8 was used to prepare the Bayesian phylogenetic analysis input file (.XML), which was analyzed in BEAST v2.7.7 to infer a time-calibrated phylogeny, producing .trees and log (.log) files. Convergence and parameter estimates were assessed in Tracer v1.7.2. The posterior tree distribution was summarized using TreeAnnotator v2.7.7 to generate a maximum clade credibility tree (.tree). Finally, the annotated phylogenetic tree was visualized and plotted in RStudio v2025.09.2 and exported as a .PNG image.

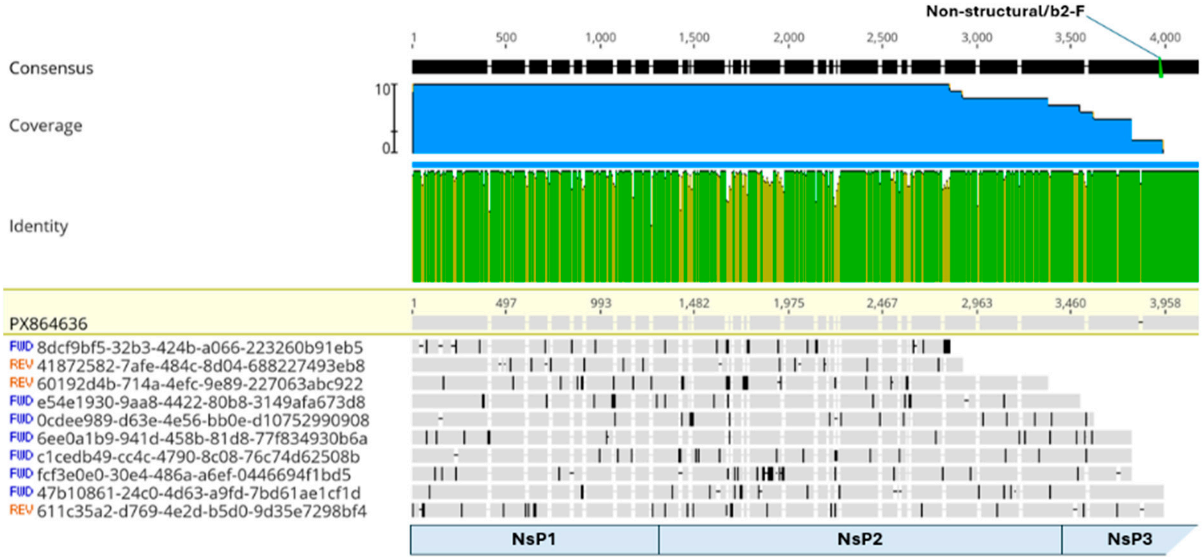

Figure S2. Read alignment, coverage, and consensus generation for the SAV2 genome sequence PX864628, representing the dataset with the lowest sequencing depth among all samples analysed. Sequencing reads from the first amplicon spanning the 5′ region of the non-structural polyprotein (nsP1–nsP3) were mapped to the SAV2 reference genome. The upper panels show the consensus sequence, coverage profile, and nucleotide identity, while the lower panel displays the individual forward and reverse reads used for consensus generation. Disagreements with consensus sequence are highlighted on aligned sequences. Although PX864628 exhibited the poorest overall sequencing performance (mean coverage 17 444, minimum coverage 2, maximum coverage 30 336), the alignment across the first amplicon showed a coherent read pattern with limited conflicting base calls and no evidence of systematic misalignment or clustered sequencing artefacts. Manual inspection of the alignment supported the consensus sequence generated for this region. Importantly, the resulting consensus was highly consistent with the corresponding genomic region of the PX864636 SAV2 sequence recovered from the same location approximately five years later, providing independent biological support for the sequence reconstruction. Together, the read-level concordance and agreement with an epidemiologically related strain indicate that the consensus sequence for the first amplicon is likely reliable despite the low and uneven sequencing depth. Regions with very low coverage should nevertheless be interpreted with caution.

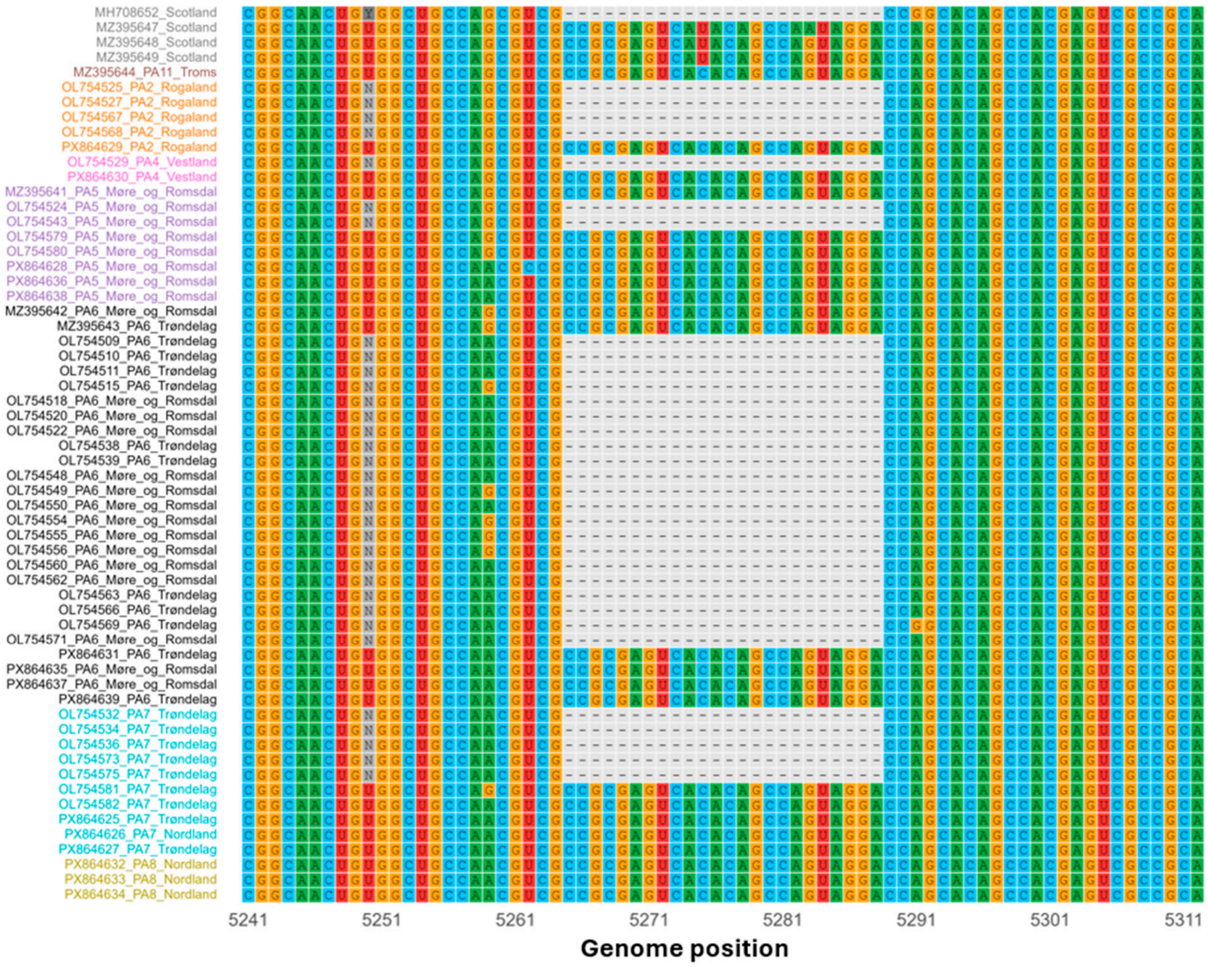

Figure S3. Alignment of the nsP3 genomic region containing a 24-nucleotide deletion in SAV2 genomes. Nucleotide alignment of SAV2 genomes spanning positions 5241–5311 relative to the reference genome MZ395648. Each row represents a single isolate and colored nucleotides indicate the aligned sequence. Gaps (–) denote the 24-nt deletion located within the nsP3 coding region. Viral samples are colored according to production area: PA2 (orange), PA4 (pink), PA5 (purple), PA6 (blue), PA7 (cyan), PA8 (green), and PA11 (brown); Scottish isolates are shown in black. The deletion is present in the majority of Norwegian SAV2 genomes across multiple production areas and years, while several Norwegian isolates and all Scottish reference genomes retain the full-length sequence. This deletion region was excluded from the Bayesian phylogenetic analyses to avoid artefactual clustering driven by the indel rather than nucleotide substitutions. The alignment illustrates the distribution of the deletion among Norwegian SAV2 lineages.

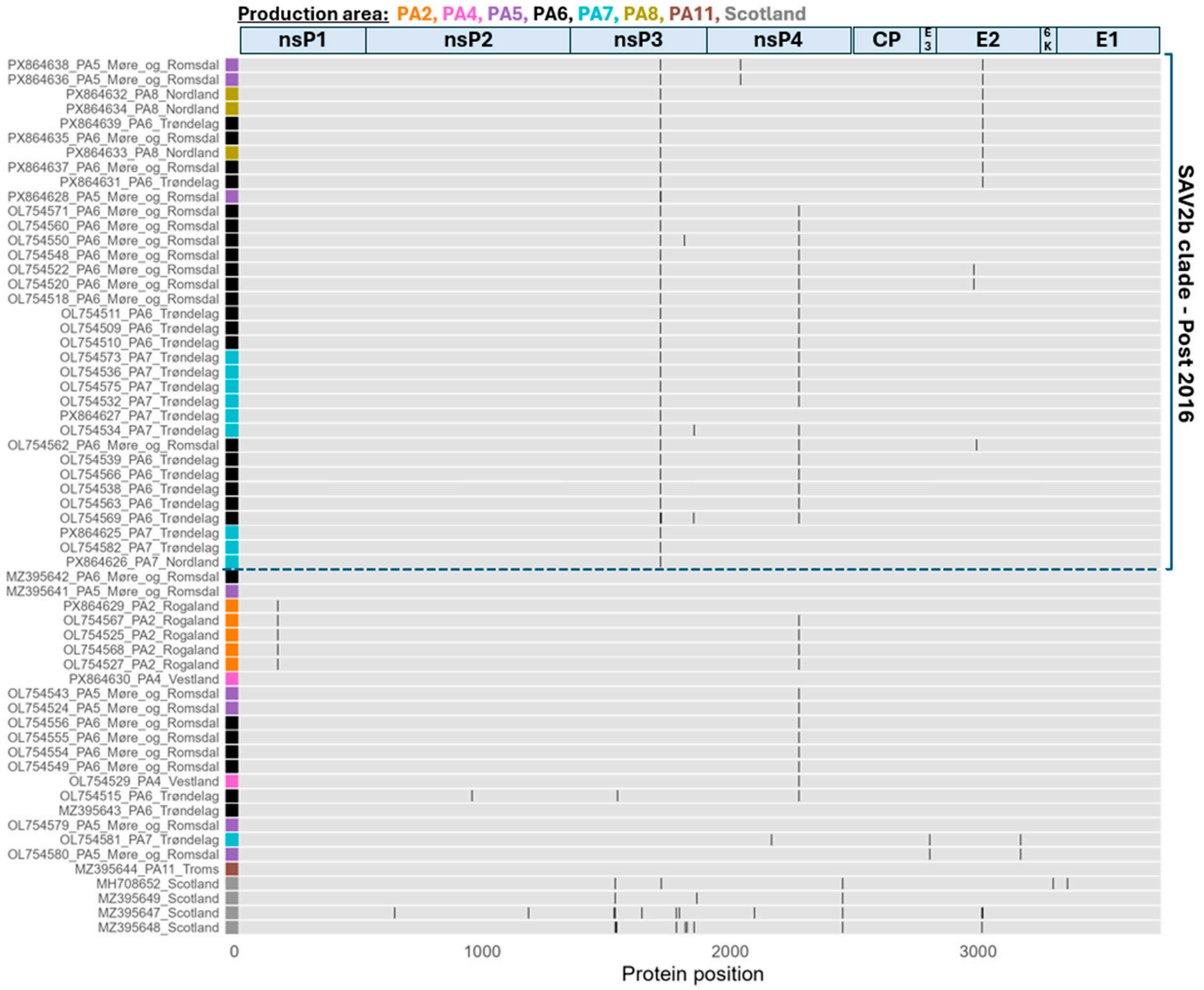

Figure S4. Amino acid variation across translated SAV2 polyproteins. Complete SAV2 genome sequences were translated and aligned relative to the reference genome MZ395644. Each row represents a single SAV2 isolate, and vertical black bars indicate amino acid positions that differ from the reference sequence. Colored bars adjacent to isolate names denote production areas as defined in legend. Protein boundaries are shown above the alignment, including the non-structural proteins (nsP1–nsP4) and structural proteins (capsid protein [CP], E3, E2, 6K, and E1). Most amino acid substitutions were observed within nsP3, whereas relatively few non-synonymous changes occurred in the remaining genomic regions. Scottish isolates (MH708652, MZ395647, MZ395648, and MZ395649), which were excluded from the Bayesian phylogenetic analysis presented in Figure 3, are included to illustrate the greater amino acid diversity observed among Scottish SAV2 genomes compared with contemporary Norwegian isolates. For simplicity, amino acids resulting from unambiguous nucleotides at position 5250 (Figure S3) are not indicated by black bars. Protein positions are shown on the x-axis.
